# A case report of Acute and transient psychotic disorder precipitated by Reiki practise

**DOI:** 10.1192/j.eurpsy.2022.2068

**Published:** 2022-09-01

**Authors:** S. Danacı, A. Asdemir

**Affiliations:** Erciyes University, Faculty Of Medicine Hospitals, Kayseri, Turkey

**Keywords:** brief psychotic disorder, Acute and transient psychotic disorders, meditation-induced psychosis

## Abstract

**Introduction:**

ATPD is defined in the ICD‐10 as a polymorphic, predominantly delusional, or schizophreniform psychotic condition characterized by an acute onset (≤2 weeks) and rapid remission (expected within 1–3 months), which is often associated with acute stressful life events. A woman in her 30s was brought to the emergency department in an acute psychotic state. Her mental health had deteriorated rapidly following her attendance to Reiki training two weeks ago (Reiki is a form of alternative medicine called energy healing). She presented as agitated, confused and had disorganised thoughts. She had paranoid, referential, misidentification and bizarre delusions.

**Objectives:**

This paper reports the case of a 37-year-old woman with stress-induced new-onset psychosis instigated by Reiki practise.

**Methods:**

A female patient is described who developed an acute and transient psychosis with polymorphic symptomatology after meditating. Physical examinations, paraclinical testing, and neuroimaging excluded an organic cause of symptoms.

**Results:**

In this case, we wanted to present an example of acute and transient psychosis episodes in which individuals with low psychosis threshold experienced recipient factors such as insomnia, dopaminergic agent (modafinil), practising reiki and meditation.While the family history of the patient, fragile personality structure suggest that the threshold of psychosis may be low; physical fatigue, insomnia, which is common in all 3 episodes, may have triggered acute psychosis.

**Conclusions:**

Our patient recovered completely within 1week after a brief admission and treatment with haloperidol. The real question here is whether the patient needs psychotropic medication for life.

**Disclosure:**

No significant relationships.

